# Assessing Awareness, Knowledge, and Attitude of Saudi Mothers Regarding Shaken Baby Syndrome in the Eastern Province of Saudi Arabia: A Cross-Sectional Prospective Study

**DOI:** 10.7759/cureus.51884

**Published:** 2024-01-08

**Authors:** Fatimah F Alghanim, Tasneem A Almubayedh, Zahra Alseba

**Affiliations:** 1 Pediatrics, Maternity and Children Hospital, Dammam, SAU; 2 Pediatric Infectious Diseases, Maternity and Children Hospital, Dammam, SAU

**Keywords:** educational interventions, preventive measures, infant brain injury, maternal awareness, shaken baby syndrome

## Abstract

Shaken baby syndrome (SBS) is a form of traumatic brain injury. Shaking babies can cause the brain matter to bounce within the cranium causing bruising and bleeding, which can result in permanent brain injury. Understanding the attitudes and knowledge of mothers on SBS would help establish effective interventions to raise awareness and establish preventive measures and education programs to avoid debilitating sequelae from SBS in newborns and infants. This study aimed to explore the awareness and attitude regarding SBS. An observational, cross-sectional study was conducted from April 1st through July 31st, 2023. The study population is comprised of mothers who are residents of the Eastern Province of Saudi Arabia and excluded females with no children and those who refused to participate, in addition to mothers not in the Eastern Province. The final sample size included 403 participants. An online-based validated questionnaire was used in the Arabic language. The questionnaire included demographic information and questions to assess the knowledge and attitude of participants regarding SBS. The chi-square test was used to test for significant associations. The majority of the participants were married (72%), while 15.6% were divorced and 10.2% were widowed. Only 7.4% of the participants were illiterates, 30.5% had primary education only, and 15.9% had postgraduate studies. Of note, 37% of the participants said that they would shake their children to calm them if they started to cry. Only 33% of the participants said that shaking babies is harmful. The most commonly reported complications of shaking babies were intracranial bleeding (48.1%), behavioral changes (23.8%), and learning disability (23.5%). Regarding attitude toward SBS, more than two-thirds (72.5%) of the participants said that they want to know more about SBS. Only the educational level had statistically significant relationship between the awareness and the sociodemographic level of the participants. This study concludes that Saudi mothers’ knowledge about SBS is inadequate despite the favorable attitude toward gaining information about it. The awareness level is significantly associated with educational status, which reflects the importance of education programs, especially during the pregnancy period, in raising awareness about SBS and its complications.

## Introduction

Shaken baby syndrome (SBS) is a form of traumatic brain injury, which occurs when a baby is shaken violently. Shaking babies can cause the delicate brain matter to bounce within the cranium causing bruising and bleeding, which can result in permanent, serious brain injuries or death. Shaken baby injuries usually occur in children under the age of two but can be seen in children up to five years old due to a heavy, large head with weak neck muscles in babies [1].

Symptoms of SBS include extreme irritability, poor feeding, breathing problems, convulsions, and pale or bluish skin [2]. While the characteristic injuries of SBS are subdural hemorrhages, retinal bleeding, damage to the spinal cord and neck, and fractures of the ribs and bones are also evident. Shaken baby injuries have a much worse prognosis [3]. Blindness can occur due to damage to the retina of the eye. The majority of babies who survive severe shaking will have some form of neurological disability, such as cerebral palsy or even mental problems, which may not be fully apparent until six years of age, and they may require lifelong medical attention [4].

A cross-sectional study done in Riyadh, Saudi Arabia, revealed that awareness of SBS was inadequate with only 32.1% of participants being aware of SBS. However, the attitude was positive among 82.5% of the participants [2]. Another study done in Tabuk City, Saudi Arabia, found that about 57.61% of their study population reported shaking the baby to make them quiet within the first year of life, 67% reported having no idea about the risks of shaking babies during the first year of life, and about 70% reported they have never heard about what is called as SBS [5].

A study conducted in Egypt on mothers after education intervention concluded that there is a positive correlation with a highly statistically significant relationship between the mother’s knowledge and behavior regarding SBS post-intervention, and the mother's awareness regarding the dangers of SBS improved after the explanation of the educational materials [6]. This indicates the influence of education intervention in improving awareness and knowledge as confirmed by another study that assessed the abusive head trauma in infants in western New York State that found that a coordinated, hospital-based, parent education program, targeting parents of all newborn infants, could reduce significantly the incidence of abusive head injuries among infants and children <36 months of age [7].

In Germany, a population-based survey revealed that 59.4% of the participants had previous knowledge about SBS. A systemic review done to assess the awareness of the public in Saudi Arabia on child abuse found that the SBS was not known to the public as a form of abuse, and the potential of developing serious injuries was not perceived by most respondents [8]. Another study from Japan found that the knowledge of the effects of shaking infants was significantly higher among women exposed to public health practices [7]. A multi-center case-control study done in New Zealand identified that the risk of abusive head trauma decreased with increasing maternal age and increasing gestational age at birth [9].

There is a lack of studies conducted in the Eastern Province of Saudi Arabia exploring the knowledge and attitude of mothers on the SBS. Understanding the attitude and knowledge of mothers on SBS in this province would help establish effective interventions to raise awareness and establish prevention measures and education programs to avoid debilitating sequelae from SBS in newborns and infants.

Objectives

This study was conducted to evaluate the awareness, knowledge, and attitude of Saudi mothers on SBS in the Eastern Province of Saudi Arabia; to determine socio-demographic factors associated with knowledge, awareness, and attitude of SBS; identify the source of knowledge of mothers about SBS; and evaluate the association between the source and knowledge level.

## Materials and methods

Study design

An observational, analytic, cross-sectional study was employed to collect data at one point in time.

Study area

This study was conducted in the Eastern Province of Saudi Arabia from April to July 2023. The Eastern Province of Saudi Arabia is the third most inhabited after Riyadh and Makkah with over 1.2 million people.

Study population

The study population is comprised of mothers who are residents of the Eastern Province of Saudi Arabia at the time of the study.

Inclusion Criteria

All Saudi mothers in the eastern providence, Saudi Arabia, with at least one child, and who consented to participate were enrolled in the study.

Exclusion Criteria

Females with no children and those who refused to participate were excluded. In addition, mothers not in the Eastern Province of Saudi Arabia were also excluded.

Sample Size

The minimum sample size (n) was calculated as follows: n=(Z2×P×Q)/D2=[(1.96)2×0.5×0.5]/0.052=384; where n=calculated sample size, Z=z-score for 95% confidence level=1.96, P=50% or the assumed proportion of participants for maximum sample size calculation, Q=(1-P)=50%, and D=the margin of error=0.05.

The minimum calculated sample size to achieve a precision of 5% with a 95% confidence interval is 384 participants and to compensate for possible non-responsiveness, we invited 403 participants

Study period

The study was conducted from April to July 2023.

Sampling technique

A convenient non-probability sampling technique was employed in this study to collect the data from the participants.

Instrument

An online-based validated questionnaire was used in the Arabic language, distributed through social media, as a Google Form targeting Saudi mothers of Eastern Province. The questionnaire included a brief description of the aim of the study and approval of participation in the first section, then demographic information and questions to assess the knowledge and attitude of participants regarding SBS. The questionnaire was designed based on the AlOmran et al. study [2].

Statistical analysis

Data was collected, then extracted using Microsoft Office Excel, then revised, coded, and fed to statistical software IBM SPSS version 22 (IBM Corp, Armonk, NY, USA) for data entry and statistical analyses. Categorical variables were expressed in frequency (percentage). Pearson’s Chi-square test was used to compare response variables. Differences were significant if the P-value is <0.05.

Ethical consideration

Respective approval of the study was obtained from the Institutional Review Board of Maternity and Children Hospital, Dammam. The ethical letter was approved on the 16th of February 2023 with ID ofPEDI-2023-0018. All participants were volunteers and asked to do their best regarding their answers. All data were kept confidential and used only for this research purpose.

## Results

A total of 403 mothers participated in the study. Out of them, 352 (87.3%) were Saudi and 47 (11.7%) were non-Saudi. Regarding their age groups, 117 (29%) were ≥40 years old, 136 (33.7%) were 30-39 years old, 120 (29.8%) were 20-29 years old, and 30 (7.4%) were <20 years old. The majority of the participants were married (72%), while 15.6% were divorced and 10.2% were widowed. Only 7.4% of the participants were illiterates, 30.5% had primary education only, 45.4% had higher education, and 15.9% had postgraduate studies. Regarding their occupational status, 44.4% of the participants were not working, while 36.7% were working and 18.1% were still studying. Nearly two-thirds of the participants (68%) described their income as being adequate. Regarding their children, 153 (38%) of the participants have one to two children, 120 (29.8%) have three to four children, 81 (20.1%) have five to six children, and 29 (7.2%) have more than six children (Table 1).

**Table 1:** Characteristics of the participants (N=403).

Characteristic	Category	Frequency	Percent
Age group	<20 years	30	7.4%
	20-29 years	120	29.8%
	30-39 years	136	33.7%
	≥40 years	117	29.0%
Nationality	Saudi	352	87.3%
	Non-Saudi	47	11.7%
Marital status	Married	290	72.0%
	Divorced	63	15.6%
	Widowed	41	10.2%
Educational level	Illiterate	30	7.4%
	Primary education	123	30.5%
	Higher education	183	45.4%
	Postgraduate studies	64	15.9%
Occupation	Working	148	36.7%
	Not working	179	44.4%
	Studying	73	18.1%
Income	Adequate	274	68.0%
	Inadequate	122	30.3%
Number of children	1-2 children	153	38.0%
	3-4 children	120	29.8%
	5-6 children	81	20.1%
	>6 children	29	7.2%

Table 2 shows the participants’ knowledge and awareness regarding SBS. Of note, 37% of the participants said that they would shake their children if they started to cry. The other usual ways used by mothers to calm their babies when they start to cry included holding their babies (63%), patting their backs (53.6%), and asking for help from family members (27%). Only 33% of the participants said that shaking babies is harmful, while 11.9% said that it is not. The most commonly reported complications of shaking babies were intracranial bleeding (48.1%), behavioral changes (23.8%), learning disability (23.5%), coma (15.1%), and blindness (12.9%). 28.8% of the participants said that there are no complications for shaking babies, while 23.1% said that it can lead to death.

**Table 2:** Knowledge and attitude regarding SBS (N=403). SBS, shaken baby syndrome

Knowledge	Category	Frequency	Percent
What do you do when your baby starts to cry?	Hold him/her	254	63.0%
	Pat his/her back	212	53.6%
	Shake him/her	151	37.5%
	Asking help from family	111	27.0%
	Not doing anything	36	8.9%
Do you think that shaking your baby is harmful	Yes	133	33.0%
	Maybe	159	39.5%
	No	48	11.9%
	Do not know	62	15.4%
In your opinion, what are the complications of shaking babies?	Blindness	52	12.9%
	Intracranial bleeding	194	48.1%
	Behavioral changes	96	23.8%
	Learning disability	95	23.5%
	Coma	61	15.1%
	There are no complications	116	28.8%
Do you think shaking babies can lead to death?	Yes	93	23.1%
	Maybe	125	31.0%
	No	183	45.4%
Attitude	Category	Frequency	Percent
Do you want to know more about SBS?	Yes	292	72.5%
	No	108	26.8%
If yes, what is your preferred source of knowledge?	Health awareness campaign	106	26.3%
	Doctors and health personnel	148	36.7%
	Internet and social media	100	24.8%
	Books and medical journals	40	9.9%
If yes, when do you prefer to know about it?	Before pregnancy	79	19.6%
	During pregnancy	171	42.4%
	After delivery	93	23.1%
	During baby’s vaccination visits	50	12.4%

Regarding attitude toward SBS, more than two-thirds (72.5%) of the participants said that they want to know more about SBS. The preferred source of knowledge was doctors and health personnel (36.7%), health awareness campaigns (26.3%), and internet and social media (24.8%). The preferred time to receive information about SBS was during pregnancy (42.4%) and after delivery (23.1%).

As shown in Table 3, only the educational level had a statistically significant relationship between the awareness and the sociodemographic level of the participants. 58% of participants with postgraduate studies knew that shaking babies is harmful, while 25.5% of participants with primary education knew that shaking babies is harmful. More information is provided in Table 3.

**Table 3:** Factors associated with knowledge about SBS (N=403). SBS, shaken baby syndrome

Characteristic	Categories	Do you think that shaking your baby is harmful			P-value
		Not harmful	Maybe	Harmful	
Age group	<20 years	5 (18.5%)	13 (48.1%)	9 (33.3%)	0.148
	20-29 years	18 (17.3%)	55 (52.9%)	31 (29.8%)	
	30-39 years	10 (8.8%)	53 (46.5%)	51 (44.7)	
	≥40 years	15 (15.8%)	38 (40.0%)	42 (44.2%)	
Nationality	Saudi	42 (14.0%)	136 (45.2%)	123 (40.9%)	0.092
	Non-Saudi	6 (16.7%)	22 (61.1%)	8 (22.2%)	
Marital status	Married	32 (12.6%)	123 (48.4%)	99 (39.0%)	0.095
	Divorced	12 (25.0%)	15 (31.3%)	21 (43.8%)	
	Widowed	3 (9.7%)	16 (51.6%)	12 (38.7%)	
Education	Illiterate	3 (15.8%)	8 (42.1%)	8 (42.1%)	0.003
	Primary education	14 (13.2%)	65 (61.3%)	27 (25.5%)	
	Higher education	24 (15.1%)	70 (44.0%)	65 (40.9%)	
	Postgraduate studies	7 (12.7%)	16 (29.1%)	32 (58.2%)	
Occupation	Working	20 (15.9%)	49 (38.9%)	57 (45.2%)	0.252
	Not working	19 (12.3%)	80 (51.9%)	55 (35.7%)	
	Studying	9 (15.9%)	29 (50.0%)	20 (34.5%)	
Income	Adequate	31 (13.4%)	107 (46.1%)	107 (46.1%)	0.926
	Inadequate	16 (15.5%)	50 (48.5%)	50 (48.5%)	
Number of children	1-2 children	15 (11.1%)	69 (51.1%)	51 (37.8%)	0.078
	3-4 children	13 (12.9%)	47 (46.5%)	41 (40.6%)	
	5-6 children	14 (21.9%)	24 (37.5%)	26 (40.6%)	
	>6 children	3 (13.0%)	7 (30.4%)	13 (56.5%)	

## Discussion

Accidental abusive head trauma includes inflicted cranial and spinal injuries that result from blunt force trauma, shaking, or a combination of these [10-12]. The resultant brain injury can be primary or secondary to the head trauma. The classic pattern that is associated with shaking babies includes diffuse unilateral or bilateral subdural hemorrhage, diffuse multilayered retinal hemorrhages, and diffuse brain injury. This pattern of injury has been previously referred to as the "shaken baby syndrome" [13,14]. The absence of a history of trauma and the external manifestations of injury can make recognition of the inflicted nature of these injuries difficult, and most of the resulting injuries can happen without the knowledge of the parents or caregivers. Therefore, in this study, we explored Saudi mothers’ awareness and attitude toward SBS and the factors that could be associated with it.

The results revealed that 37.5% of the mothers shake their babies to calm them if they start to cry. This indicates that many of the participants do not know about the serious complications that can result from shaking their children. In addition, about one-third of the participants (33%) thought that shaking their babies is harmful, while another 39.5% of them said it might be harmful. 

Our results are close to what was reported by other Saudi studies, for example, a recent 2023 study conducted in Riyadh reported that one-third of participants (30%) knew about SBS [15]. Another study by AlOmran et al. also reported that 33.6% of participants thought that shaking babies is harmful and 41.9% were unsure of it [2]. Also, according to a survey conducted in Tabuk City in 2018, 67.39% of participant parents were unaware of the dangers of shaking babies, and in Makkah, it was noticed that most participants had never heard about SBS and those who were aware of it their sources were not reliable or trusted [5,16]. Furthermore, a survey conducted in Egypt reported that the majority of mothers (80%) did not know about SBS, while only 20% did [6]. In addition, the Mann et al. study showed that 54% of individuals were unaware of this syndrome [17]. More than 57% of the participants, according to Marcinkowska et al., have heard of the syndrome [18].

These findings indicate a low level of awareness regarding SBS, which could be due to the fact there are not enough educational programs targeting mothers about such topics. Although many parents do shake their children, the majority do not perceive this practice to be harmful.

Our results also found that the most commonly reported complications of SBS were intracranial bleeding (48.1%), behavioral changes (23.8%), learning disability (23.5%), coma (15.1%), and blindness (12.9%). It is worth mentioning that 28.8% of the participants said that there are no complications for shaking babies, while 23.1% said that it can lead to death. According to the AlOmran study, the reported complications of shaking babies included cerebral hemorrhage (41%), behavioral changes (39.3%), and learning disability (27.2%); while 28.8% thought that it may lead to death [2]. These results, which are similar to our study findings, are due to the fact that both studies were conducted in Saudi Arabia. According to the literature, injuries associated with shaking babies include retinal hemorrhages, intracranial bleeding, skull fractures, and other injuries [11]. It is worth mentioning that significant intracranial bleeding rarely occurs as a result of shaking babies, and several studies confirm the rarity of serious intracranial injury [19].

The result of this study shows that educational level has a significant association with knowledge about SBS. Education of the mothers may help to prevent inappropriate treatment of crying babies. This is highlighted by the result of an educational program, which was conducted in Saudi Arabia in 2014 where a pilot awareness program done initially reported that 77% of their participants had little to no knowledge of SBS risk factors and complications. However, after six months, the participants could recall at least 50% of the educational program [20]. AlOmran et al. found that gender, marital status, and occupation were significantly associated with awareness level [2].

Regarding participants‘ attitudes, more than two-thirds (72.5%) of the participants said that they want to know more about SBS. Multiple Saudi studies highlighted this favorable attitude toward this issue and indicated the willingness of Saudi mothers to participate in educational programs or other methods to raise their awareness about SBS [2,15]. According to our participants, they preferred to take knowledge about SBS from doctors and health personals (36.7%), followed by health awareness campaigns (26.3%), and fewer participants preferred to take knowledge from the internet and social media (24.8%). This is inconsistent with AlOmran et al. where the preferred method of learning was online webpages and not with direct contact with doctors. The preferred time to receive this information was during pregnancy (42.4%) and after delivery (23.1%); Mann et al. also reported a similar preferred timing of learning, which was during the pregnancy period [17].

Limitation

The study in Saudi Arabia's Eastern Province reveals a lack of understanding of SBS among mothers, with 37.5% admitting to shaking babies to quiet them. Despite a positive attitude toward learning more about SBS (72.5%), there is a considerable information gap, which is particularly associated with educational levels. The study underlines the importance of focused educational efforts during pregnancy to raise awareness and minimize newborn damage.

## Conclusions

This study concludes that Saudi mothers’ knowledge about SBS is inadequate despite the favorable attitude toward gaining information about it. The awareness level is significantly associated with educational status, which reflects the importance of education programs, especially during the pregnancy period, in raising awareness about SBS and its complications.
